# Comparison of visual and objective quantification of elbow and shoulder movement in children with obstetric brachial plexus palsy

**DOI:** 10.1186/1749-7221-1-5

**Published:** 2006-12-01

**Authors:** Andrea E Bialocerkowski, Mary Galea

**Affiliations:** 1 Rehabilitation Sciences Research Centre, School of Physiotherapy, The University of Melbourne, VIC, 3010, Australia

## Abstract

**Background:**

The Active Movement Scale is a frequently used outcome measure for children with obstetric brachial plexus palsy (OBPP). Clinicians observe upper limb movements while the child is playing and quantify them on an 8 point scale. This scale has acceptable reliability however it is not known whether it accurately depicts the movements observed. In this study, therapist-rated Active Movement Scale grades were compared with objectively-quantified range of elbow flexion and extension and shoulder abduction and flexion in children with OBPP. These movements were chosen as they primarily assess the C5, C6 and C7 nerve roots, the most frequently involved in OBPP. Objective quantification of elbow and shoulder movements was undertaken by two-dimensional motion analysis, using the v-scope.

**Methods:**

Young children diagnosed with OBPP were recruited from the Royal Children's Hospital (Melbourne, Australia) Brachial Plexus registry. They participated in one measurement session where an experienced paediatric physiotherapist facilitated maximal elbow flexion and extension, shoulder abduction and extension through play, and quantified them on the Active Movement Scale. Two-dimensional motion analysis captured the same movements in degrees, which were then converted into Active Movement Score grades using normative reference data. The agreement between the objectively-quantified and therapist-rated grades was determined using percentage agreement and Kappa statistics.

**Results:**

Thirty children with OBPP participated in the study. All were able to perform elbow and shoulder movements against gravity. Active Movement Score grades ranged from 5 to 7. Two-dimensional motion analysis revealed that full range of movement at the elbow and shoulder was rarely achieved. There was moderate percentage agreement between the objectively-quantified and therapist-rated methods of movement assessment however the therapist frequently over-estimated the range of movement, particularly at the elbow. When adjusted for chance, agreement was equal to chance.

**Conclusion:**

Visual estimates of elbow and shoulder movement in children with OBPP may not provide true estimates of motion. Future work is required to develop accurate, clinically-acceptable methods of quantifying upper limb active movements. Since few children attained full range of motion, elbow and shoulder movement should be monitored and maintained over time to reduce disability later in life.

## Background

Obstetric brachial plexus palsy (OBPP) is a complication of childbirth, which is characterized by one or more nerve conduction blocks within the brachial plexus [1]. These blocks range in severity and location within the plexus and primarily affect the child's ability to move and effectively use their affected upper limb [2]. Thus the quantification of motor function is essential when assessing children with OBPP [3].

The assessment of motor function in young children is more difficult when compared with adolescents and adults [4]. Young children often lack cooperation and communication skills [5]. Thus they experience difficulty following commands to move or maintain their limbs in test positions for measurement [6]. Measurement of the child's ability to move their affected upper limb is further complicated by spontaneous, rapid movements that often occur in infants and young children [7]. As such, in the clinical setting active range of movement of the child's affected limb is infrequently measured with a goniometer or inclinometer. Rather it is facilitated by play, visually estimated and usually quantified on a rating scale [8].

One such rating scale for the quantification of movement in children with OBPP is the Active Movement Scale. Developed by Clarke and Curtis [8] it evaluates overall joint movements, such as shoulder flexion and elbow extension, in positions where gravity is eliminated and against gravity. Movement is quantified on an eight-point ordinal scale, with 0 equating to "no contraction visible" and 7 being "full motion" present (Table 1). This measurement tool has moderate to excellent intra- and inter-rater reliability when used by experienced clinicians on children with OBPP between 1 month to 15 years of age [4,9].

**Table 1:** The Active Movement Scale [15]

Observation	Muscle grade
Gravity eliminated	
• No contraction	0
• Contraction, no motion	1
• Motion ≤ 1/2 range	2
• Motion > 1/2 range	3
• Full motion	4
Against gravity	
• Motion ≤ 1/2 range	5
• Motion > 1/2 range	6
• Full motion	7

One advantage of using this rating scale is that is quantifies movement in categories, such as "motion greater than half range". This procedure theoretically produces less variability in scoring and may provide higher reliability coefficients and smaller measurement errors compared to direct measurement of active movement [10]. However, no comparison has been made between therapist-rated Active Movement Scale grades and objectively-quantified range of active movement. This is important to determine as management decisions are based on Active Movement Scale grades [3] and currently the accuracy of the Active Movement Scale is not known. This study addressed this gap in the evidence by comparing therapist-rated Active Movement Scale grades with objectively-quantified range of elbow flexion and extension and shoulder abduction and flexion in children with OBPP. These movements were chosen as they primarily assess the C5, C6 and C7 nerve roots, which are the most frequently involved in OBPP [11]. Objective quantification of elbow and shoulder movements was undertaken by two dimensional motion analysis, using the v-scope.

## Methods

Data collection was part of a larger study which investigated the intra- and inter-rater reliability of two-dimensional motion analysis (using the v-scope [Eshed Robotics Inc]) to quantify elbow and shoulder movement in young children with OBPP. Detailed information regarding the v-scope and the method of movement quantification can be found in another publication [12]. Prior to the commencement of the study, ethical approval was gained from the Royal Children's Hospital, Melbourne, Australia and The University of Melbourne, and consent was gained from the participating families.

### Participants

All families of children on the OBPP registry at the Royal Children's Hospital, aged between six months and four and a half years were eligible to participate in this study, irrespective of their functional status, residential location or method of management. Case notes were used to confirm the diagnosis of OBPP and to gather demographic information about the child.

### Objective quantification of active movement

The v-scope, a two-dimensional motion analysis system was used to quantify the maximal range of elbow flexion and extension and shoulder abduction and flexion. Its three transmitting towers were configured in an L-shape and they located the position of up to four "buttons" which were placed by an experienced paediatric physiotherapist on standardized landmarks on the child's affected upper limb, trunk and chest. The location of these "buttons" was based on a pilot study of 10 non-impaired children. Each child was positioned on the floor in the centre of the towers' fields at a distance of 1.5 meters from the towers.

The same, experienced paediatric physiotherapist facilitated the maximal range of elbow flexion and extension, shoulder abduction and flexion through play or by tapping the corresponding muscle group, according to the procedures outlined by Curtis et al [9]. Three repetitions of each of the movements were conducted in a standardized order. This information was captured by the v-scope. In addition, the same paediatric physiotherapist quantified each movement on the Active Movement Scale.

### Data analysis

Demographic characteristics of the participants and the maximal range of elbow flexion and extension, shoulder abduction and flexion were summarised using descriptive statistics. Data were extracted from the v-scope program and exported into Microsoft Excel 2005. Angles that depict the maximum range of elbow flexion and extension, shoulder abduction and flexion were calculated by dot product from the vectors for adjacent segments, which were gained from the X, Y and Z co-ordinates of the "buttons". This produced values, in degrees, for maximal elbow flexion and extension, shoulder abduction and flexion. Three maximal angles for each movement were subsequently identified, averaged and used in all analyses. As three Active Movement Scale grades were generated for each direction of movement, the mode was used in all analyses. Analyses revealed that the Active Movement Score did not change between repetitions for any of the movements.

To determine the accuracy of the therapist-rated Active Movement Score grades for each direction of movement, therapist-rated grades were compared with objectively-quantified Active Movement Score grades. Objectively-quantified Active Movement Score grades were generated by collapsing the maximal angles gained by the v-scope, measured in degrees, into the appropriate categories on the Active Movement Scale. This required knowledge of what constitutes half range of elbow flexion and extension, shoulder abduction and flexion. Normative values described by Boone et al [13] were subsequently used as they were established in children of a similar age group. As range of motion is variable in subjects with "normal" elbows and shoulders, "full motion" was defined as motion that was greater than or equal to the lower 95% confidence point of maximal range. This motion was halved to determine the cut off point between grades of movement, such as grade 5 (Motion ≤ 1/2 range) and grade 6 (Motion > 1/2 range) (Table 2). Percentage agreement and Kappa statistics, which correct for chance agreement, were calculated to determine the agreement between the two sets of Active Movement Scores [14]. All analyses were conducted in Statistical Packages for Social Sciences (SPSS) (Version 13). Where Kappa values could not be calculated in SPSS, due to the lack of spread of data, they were calculated by hand using the formula outlined in Portney and Watkins [15].

**Table 2:** Normative values and cut off points for conversion of angular into Active Movement Scores

		Active Movement Score cut-offs		
		
Direction of movement	Norm* – Maximal range (95% CI)	Motion ≤ 1/2 range	Motion > 1/2 range	Full motion
Elbow flexion	145.4° (144.0°–146.8°)	≤ 71.1°	71.2–143.9°	≥ 144.0°
Elbow extension	0.6° (0.1°–1.7°)	≥ 71.2°	1.8°–71.1°	≤ 1.7°
Shoulder abduction	185.4° (184.4°–186.4°)	≤ 92.1°	92.2°–184.3°	≥184.4°
Shoulder flexion	168.4° (167.4°–169.4°)	≤ 83.6°	83.7–167.3°	≥ 167.4°

* Boone et al [13], CI confidence interval

## Results

Our sample consisted of 30 children with OBPP (18 females, 12 males), aged between 0.6 and 4.6 years. Most participants were diagnosed with a C5, C6 (43%) or C5, C6, C7 (37%) nerve conduction block and few underwent a primary nerve repair within the first year of life (n = 4). None of the participants had undergone secondary shoulder or elbow surgery.

The average range of elbow flexion and extension, shoulder abduction and flexion is presented in Table 3. When these values are compared to the norms in Table 2, it can be seen that very few participants' movement could be considered as "full" or "normal". Only five participants gained what was considered "full motion" for elbow extension, and none of the participants recorded "full motion" for elbow flexion, shoulder abduction and flexion. All subjects were able to perform the elbow and shoulder movements against gravity. Thus their movement was graded as 5, 6 or 7 on the Active Movement Scale.

**Table 3:** Range of elbow and shoulder movement in children with obstetric brachial plexus palsy

Direction of movement	Mean (95% CI)	Range	n
Elbow flexion	115.2° (108.1–122.3°)	69.7°–143.3°	29
Elbow extension	10.5° (6.6°–14.4°)	1.0°–52.0°	30
Shoulder abduction	116.7° (108.1°–125.3°)	62.3°–158.3°	27
Shoulder flexion	138.2° (135.1°–141.3°)	116.7°–157.0°	30

CI confidence interval, n number of participants

Table 4 illustrates the frequency of therapist-rated and objectively-quantified Active Movement Scale grades. There was moderate percentage agreement in Active Movement Scale grades, which ranged from 41% (elbow flexion) to 70% (shoulder flexion). When this was corrected for chance agreement, agreement was equal to chance. Movement was most frequently over-estimated by the paediatric physiotherapist, at the elbow more than at the shoulder.

**Table 4:** Comparison of the therapist-rated and objectively-quantified Active Movement Score grades

		Frequency		Agreement	
		
Direction of movement	Active Movement Scale Grade	Therapist-rated	Objectively-quantified	% agreement	Kappa
Elbow flexion	5	3	1		
	6	13	28	41	-0.05
	7	13	0		
Elbow extension	5	0	0		
	6	10	25	43	0.07
	7	20	5		
Shoulder abduction	5	5	4		
	6	17	23	56	0.0
	7	5	0		
Shoulder flexion	5	3	0		
	6	21	30	70	0.0
	7	6	0		

## Discussion

This is the first known study that has compared therapist-rated Active Movement Scale grades with objectively-quantified active range of elbow flexion and extension and shoulder abduction and flexion in children with OBPP. We found that there was little agreement in the muscle grades, with the paediatric physiotherapist most frequently over-estimating the movement gained. This discrepancy was most apparent between grades 6 and 7, when the therapist graded the movement as "full motion" (grade 7) when it was less than what is considered full movement in children with "normal" elbows and shoulders. This more frequently occurred at the elbow rather than at the shoulder.

Overestimation of range of elbow flexion and extension may have occurred due to error in interpreting what constitutes half range at this joint. Ninety degrees of flexion at the elbow tends to be easy to visualize as the forearm is horizontal when the subject is in the sitting position [16]. This is often what is thought to guide the quantification of range of movement at the elbow. However, 90° of elbow flexion does not represent half range of motion as full motion of the elbow is typically 140°–150° [13,17]. Thus it may be difficult to judge when the elbow is at 70°, which is 20° before the forearm is horizontal. This is in contrast to the shoulder, which has 165°–185° of flexion or abduction [13,18]. Half range of motion is at approximately 90° of flexion or abduction, ie when the forearm is in the horizontal position.

Our choice of norms may also have contributed to the lack of agreement between the two sets of Active Movement Scale grades. Currently, there is little evidence regarding what is "normal" range of movement at the elbow and shoulder in young children. One hundred and twenty degrees has been used as "normal" range of movement at the elbow by clinicians treating children with OBPP [19]. However there is no evidence to support this contention. In this study, we used Boone et al's [13] norms, as they were developed on a sample that most closely matched the one used in this study. Despite this, there were differences between the two samples and methodologies that should be considered when interpreting our results. For example, 1–19 year old males were used in Boone et al's [13] study, compared with 0.5–4.6 year old children (18 female, 12 male) in the present study. Range of movement decreases with age [13,18] and females tend to have greater range of movement compared with males [18]. However it is not known whether these changes take place before or after puberty or later in life. Although hand dominance does not to influence shoulder movement, Gundal et al [17] found that there were significant differences in elbow movement between the right and left sides (differences of approximately 2°). Boone et al [13] did not produce left and right sided norms for the elbow and shoulder.

A hand-held goniometer was used to produce the norms by Boone et al [13]. This measurement tool may have a greater measurement error compared to using two-dimensional motion analysis to quantify elbow and shoulder movement in young children. Measurement error for the v-scope (2× standard error of measurement) was less than 15° [12]. However measurement error for hand-held goniometry in young children has not been established. Considering the rapid, spontaneous upper limb movement in young children, measurement error may be considerable in this population [7]. Thus future studies are required to determine the range of "normal" elbow and shoulder movement in young children using various methods, which are feasible to use in the clinical setting.

Despite these limitations, it is acknowledged that there is error associated with all types of measurements. However, the aim of clinical measurement is to reduce this error as much as possible during assessment and to consider the degree of measurement error when interpreting the results [15]. Developers of the Active Movement Scale sought to reduce variability in scoring by quantifying movement in categories [8]. Although this is theoretically sound [10], the Active Movement Score grades may not represent true, objectively-quantified movement at the elbow and shoulder. Thus, future work is required to develop accurate, clinically acceptable methods of quantifying upper limb active movements.

We also found that children with OBPP rarely gain full range of elbow flexion and extension, shoulder flexion and abduction. Lack of elbow and shoulder range of movement may compromise the ability to perform daily tasks [20]. Since range of shoulder and elbow movement decrease with age [13,18] and the symptoms of OBPP are exacerbated with age and produce increasing disability [20], our results provide a justification to monitor and maintain upper limb movement in children, adolescents and adults with OBPP.

## Conclusion

The main finding of this study is that visual estimates of elbow and shoulder movement in children with OBPP may not provide true estimates of motion. Since decisions regarding the optimal management strategies, including whether surgery is indicated, are often made based on this type of assessment, clinicians should interpret their results with care. Moreover future work is required to develop accurate, clinically-acceptable methods of quantifying upper limb active movements. A secondary finding was that few children attained full range of motion. Hence, elbow and shoulder movement should be monitored and maintained over time to reduce disability in adolescence and adulthood.

## Competing interests

The author(s) declare that they have no competing interests.

## Authors' contributions

AB participated in the study design, preparation of the ethics application, trained research assistants, monitored the project for quality, entered data, data analysis and manuscript preparation. MG participated in the study design, preparation of the ethics application and writing of the manuscript.
